# Effect of Addition of Green Coffee Parchment on Structural, Qualitative and Chemical Properties of Gluten-Free Bread

**DOI:** 10.3390/foods10010005

**Published:** 2020-12-22

**Authors:** Paola Littardi, Massimiliano Rinaldi, Maria Grimaldi, Antonella Cavazza, Emma Chiavaro

**Affiliations:** 1 Dipartimento di Scienze degli Alimenti, Università degli Studi di Parma, Parco Area delle Scienze 47/A, 43124 Parma, Italy; paola.littardi@unipr.it (P.L.); massimiliano.rinaldi@unipr.it (M.R.); 2 Dipartimento di Scienze Chimiche, della Vita e della Sostenibilità Ambientale, Università degli Studi di Parma, Parco Area delle Scienze 17/A, 43124 Parma, Italy; maria.grimaldi@unipr.it (M.G.); antonella.cavazza@unipr.it (A.C.)

**Keywords:** green coffee, parchment, gluten-free bread, quality, antioxidant, total phenols, physico-chemical properties

## Abstract

Green coffee parchment (GCP) is becoming interesting, due to the diffusion of wet processing in which coffee parchment is collected separately; it is one of the less studied coffee by-products, but it is reported to be rich in phenolic compounds and dietary fiber. The addition of GCP (355–500 μm) at 2% to gluten-free breads was investigated in terms of physical properties (volume, moisture content, water activity, crumb grain, texture, and color), total antioxidant capacity (TAC) and total phenol content during three days of storage. Moreover, the effects of GCP on sensorial characteristics, 5-hydroxymethylfurfural (HMF), and oxidative stability was evaluated. From the sensorial analysis, bread with 2% addition resulted in being acceptable for consumers with no significant differences from the control, while 4% of GCP was discarded by consumers, as it resulted in being too bitter. Moreover, GCP at 2% addition did not modify volume, moisture content, and water activity. On the contrary, GCP deeply affected the color with a darker aspect that was appreciated by consumers. Regarding texture, 2% of GCP did not affect hardness, cohesiveness, and staling process during storage. Interestingly, 2% of GCP significantly improved the TAC and oxidative stability of the bread; in accordance with these results, 2% of GCP reduced the HMF content, thanks to its antioxidant compounds.

## 1. Introduction

Global green coffee (*Coffea Arabica* and *Coffea canephors*) production resulted in 10.3 Mtons in 2018 [1], with Brasil (3.6 Mtons), Vietnam (1.6 Mtons), Indonesia (0.7 Mtons), and Colombia (0.7 MTons) as the main producers. The fruit of coffee is composed of different anatomical parts (skin, pulp, pectin layer, parchment, silverskin, and beans) with different morphology and characteristics. To date, coffee beans are almost exclusively used for the preparation of the brew or beverage, obtained by the infusion of roasted and ground beans [2]. Considering coffee as the first worldwide food commodity [3], the extent of waste deriving from its chain can be understood. The whole fruit of coffee is rich in fiber, phytochemical compounds, and nutrients, but 90% of them are lost during processing in waste or by-products [2]. In recent years, an important challenge for food industries and food researchers is enhancing and reusing each by-product on the basis of its functional properties. Among coffee by-products, silverskin represents the most studied one: for example, Pourfarzad et al. [4] used treated coffee silverskin for the enhancement of Barbari flat bread in terms of reduction of caloric intake and increase of dietary fiber content. Iriondo-De Hond et al. [2] suggested the use of coffee husks for its antioxidant and dietary fiber content. Similarly, Gocmen et al. [5] evaluated the addition of coffee silverskin to improve the functional and nutritional properties of cookies. Among the components of the green fruit parchment, a fibrous endocarp that wraps and separates the seeds of coffee, represents ~6.1% of the weight of the entire fruit [3,6], and its reuse was partially studied. The parchment content in phenolic compounds is reported to be 1.2–3.1 mg g^−1^ GAE [7], while its fiber content is about 92% [2]. Cubero-Castillo et al. [6] studied coffee parchment-enriched biscuits and observed that 2% of supplementation was well accepted by panelists, and biscuits showed 5.4% of fiber content and an antioxidant activity of 1116 μmol TE/100 g. Moreover, parchment was suggested as an antifungal additive with potential uses for food preservation [8]. Finally, the safety of coffee parchment was demonstrated by the absence of pesticides and mycotoxins (aflatoxin B1 and enniantin B) [9].

Coffee parchment is a by-product of coffee wet processing, in which the coffee parchment is removed after drying and hulling in distinct steps, which permits its collection and use separately from other by-products [3]. By considering that approximately 40% of all coffee around the world is wet processed, because it is considered to produce superior quality [10], coffee parchment represents an important waste and deserves valorization.

In the last decades, with the growing number of people affected by celiac disease, the market increased the call for gluten-free bakery products, that are known to be intrinsically poor in fiber [11]. Aqueous extracts of coffee silverskin and husk were added in gluten-free bread to obtain healthier products [12]. In this direction, the use of green coffee parchment could be an interesting solution to improve both the fiber and nutraceuticals content of gluten-free bread. On the other hand, it is well known that the addition of dietary fiber from different origins could affect the quality characteristics of the final product [13]. For these reasons, the aim of this study is to evaluate, for the first time in the literature, the addition of ground green coffee parchment to gluten-free bread, considering the physical, physico-chemical, and sensory properties during shelf life.

## 2. Materials and Methods

### 2.1. Green Coffee Parchment Characteristics

The moisture, protein, lipid, and ash of green coffee parchment were determined using standard procedures [14]. Moisture content was determined in the oven at 105 °C for 24 h. Crude fat content (AOAC, Method 920.39) [14] was determined with diethylether, using an automatized Soxhlet extractor (SER 148/3 VELP SCIENTIFICA, Usmate Velate, Italy). Total ash was determined in a muffle furnace at 550 °C for 10 h (AOAC, Method 923.03) [12]. Total nitrogen (AOAC, Method 955.04) [14] was determined with a Kjeldahl system (DKL heating digestor and UDK 139 semiautomatic distillation unit, VELP SCIENTIFICA), while fiber was determined with the gravimetric method (AOAC, Method 7.504) [14].

Water binding capacity (*WBC*), water absorption index (*WAI*), water solubility index (*WSI*), and water holding capacity (*WHC*) were determined on green coffee parchment [15]. *WBC* is defined as the amount of water retained by the sample under low-speed centrifugation: 1.000 ± 0.005 g of samples were mixed with distilled water (10 mL) and centrifuged at 2000× *g* for 10 min. *WAI* or swelling capacity and *WSI* were determined on 50.0 mg ± 0.1 mg of green parchment samples dispersed in 1.0 mL of distilled water and cooked at 90 °C for 10 min in a water bath under stirring. The cooked paste was cooled in an ice water bath for 10 min, and then centrifuged at 3000× *g* at 4 °C for 10 min. The supernatant was decanted into an evaporating dish and the weight of dry solids was recovered by evaporating the supernatant at 105 °C till constant weight. Pellets (*Wr*) and dried supernatants (*Ws*) were weighed and *WSI* or *WAI* were calculated as follows:

(1) WSI(gg)=WsWi

where *W_i_* was the sample weight.

*WHC* is defined as the amount of water retained by the sample without being subjected to any stress: 1.000 ± 0.005 g of samples were mixed with distilled water (10 mL) and kept at room temperature for 24 h. The supernatant was decanted. The results were expressed as grams of water retained per gram of solid, as the average of three replicates.

The pH of the parchment was determined by means of triplicate measurements with a pH meter (Jenway, Staffordshire, UK) on parchment/distilled water suspension at a 1:10 ratio [14].

### 2.2. Bread Samples Preparation and Storage

A commercial (Dr. Schär AG/SPA, Burgstall (BZ), Italy) gluten-free bread mixture was purchased at a local supermarket and showed the following ingredients on the product label: corn starch, rice flour, vegetable fibers (psyllium, bamboo), whole rice flour (3.8%), lentil flour (3.6%), dextrose, thickening agent (hydroxypropylmethylcellulose), and salt. The proximate composition on 100 g of the mixture was: moisture 11.2 g, carbohydrates 80 g (sugars 1.4 g), fat 0.9 g (saturated 0.2 g), fibers 4.4 g, proteins 2.7 g, and salt 0.8 g. The ground green coffee parchment (P) was kindly donated by the company Ferri dal 1905 (Castel Goffredo, MN, Italy) and a particle size in the range 355–500 μm was obtained by means of mechanical sieving.

Control bread samples (C) were prepared with the following recipe: gluten-free bread mixture (250.0 g), tap water (200.0 g), sunflower oil (10.0 g), compressed brewer’s yeast (5 g), and salt (2.5 g). All the ingredients were purchased from a local supermarket. The recipe was developed starting from the instructions reported on the label of the gluten-free mixture with little adjustments for obtaining an acceptable product with the bread machine used in this work.

Parchment breads (PB) were produced, substituting 2% (corresponding to 5 g) of the mixture with P. Higher levels of substitution were not analyzed because previous consumer acceptance tests revealed that substitution at 4% conferred a marked bitter flavor to the final product, maybe due to the presence of caffeine: as caffeine content in the coffee parchment was reported to be about 58 mg/g [2], with 10 g of P, the concentration of caffeine will be 1.6 mg/g of bread.

A domestic bread machine (Moulinex, Groupe Seb Italia S.p.A., Milano, Italy) was used for breadmaking, with a rapid program: stirring + kneading + rising, 80 min; baking, 55 min at 210 °C. Samples were then cooled down at room temperature for at least 2 h and packaged in alcohol-sprayed sealed air-tight plastic bags, stored in the dark at 25 °C in a temperature-controlled chamber (ISCO 9000, Milan, Italy) and analyzed at 0, 1, and 3 days (D_0_, D_1_ and D_3_) of shelf life. Three batches were produced and analyzed for each bread formulation.

### 2.3. Bread Physical Analyses

Specific volume (V, mL/g) of breads was determined by rapeseed displacement method in triplicate according to the AACC Approved Method 10–05.01 [16].

Moisture content (MC, g/100g) of the bread crumb was determined in triplicate on each sample by drying at 105 °C to constant weight AACC standard method, 44–15.02 [16]. Water activity (*a*_w_) of crust and crumb was measured at 25 °C (AQUALAB, Decagon Devices Inc., Pullman, Washington, DC, USA). Three replicates for each batch per sample were measured.

Crumb grain was evaluated by means of a digital image analysis system, as previously reported [17]. Images of 3 central slices (thickness 20 mm) of each loaf were acquired with a scanner (Hewlett Packard, Palo Alto, CA, USA) at 600 dots per inch (dpi) taking squares of 40 × 40 mm from the center of the images after calibration, standardization, and optimization by means of ImageJ (National Institutes of Health (NIH), Maryland, USA) software. The number of pores (expressed as percentage of the total number) was obtained according to four pre-selected dimensional classes (cl.1: 0.01–0.099 mm^2^; cl.2: 0.1–0.199 mm^2^; cl.3: 0.2–0.99 mm^2^; cl.4: >1 mm^2^).

Texture analysis was performed on the crust and crumb using a TA.XT2 Texture Analyzer equipped with a 25 kg load cell (Stable Micro Systems, Godalming, UK) and Texture Expert for Windows software (version1.22) for data analysis on each loaf. The maximum peak force obtained from the crust puncture test (P3 stainless steel probe, speed of 2 mm/s) was taken from the penetration curve on the whole loaf and considered as hardness (N). Crumb evaluation was carried out with a TPA test (P/35 cylindrical aluminum probe, speed of 2mm/s up to the 40% of the original sample height) on ten cubes of 20 × 20 × 20 mm extracted from two central slices of the samples. The textural parameters considered were hardness (maximum peak force of the first compression cycle, N), cohesiveness (ratio of positive force area during the second compression to that during the first compression area, dimensionless), springiness (ratio of the length of the second to the first compression peak), resilience (area during the withdrawal of the penetration, divided by the area of the first penetration, dimensionless), and chewiness (product of hardness x cohesiveness x springiness, N) [18].

Color was determined on fifteen pre-selected locations of the crust and crumb of each bread loaf. The analyses were performed using a Minolta Colorimeter (CM 2600d, Minolta Co., Osaka, Japan) equipped with a standard illuminant D65 and a 10° position of the standard observer. L* (lightness), a* (redness) and b* (yellowness) were quantified on each sample using the Spectramagic software (Ver. 3.6) after calibration with white and black tiles.

### 2.4. Bread Chemical Analyses

#### Chemicals

Water (MilliQ), ethanol 96%, methanol 99%, acetone, sodium carbonate, Folin–Ciocalteu reagent, Carrez I and Carrez II reagents, DPPH (2,2-diphenyl-1-picrylhydrazyl), 5-hydroxymethylfurfural, and gallic acid analytical standard were all purchased from Sigma-Aldrich.

### 2.5. Total Phenolic Content (TPC), Total Antioxidant Capacity (TAC), and Oxitest

Of the sample, 1.2 g were added to 20 mL of acetone, sonicated for 30 min, and centrifuged at 24 °C and 6000 rpm for 10 min. The supernatant was collected in a flask: this operation was repeated three times ensuring a complete extraction. The extract was dried under a gentle nitrogen stream, then 10 mL of ethanol were added, and the solution was filtered through a PTFE filter 0.45 µm × 25 mm.

For the evaluation of the total phenolic content (TPC), 50 μL of sample extract were added to 1160 μL of water (MilliQ), 300 μL of sodium carbonate 20% *w/w*, and 100 μL of the Folin–Ciocalteu reagent; the solution was then incubated at 40 °C for 30 min [13]. An identical preparation was performed in the absence of the sample for blank evaluation. Absorbance was measured at 760 nm on a UV-vis Spectrophotometer (Thermo Scientific™ Evolution™ 201/220). The TPC value was expressed as µg of GAE (gallic acid equivalent)/g of dry sample. The calibration curve was built using gallic acid in the concentration range from 0.78 to 25 µg/mL. All analyses were performed in triplicate.

The total antioxidant capacity (TAC, %) was determined following the method previously reported [19]. An amount of 500 μL of the sample extracts, prepared as for total phenolic compounds determination, was added to 1.5 mL of freshly-prepared 60 μM 2,2-diphenyl-1-picrylhydrazyl (DPPH) radical solution in methanol. After 30 min, the absorbance of the solution was measured at 517 nm. The observed value was compared to that of the DPPH radical solution in methanol and read at the same wavelength, but at t = 0 min.

The oxidative stability was measured by Oxitest (Velp Scientifica, MB, Italy) at the following operative conditions: 30 g of minced bread were placed in the reactor chamber at the temperature of 90 °C and oxygen pressure of 6 bar. Measures were performed in duplicate.

### 2.6. Determination of 5-HMF

High Performance Liquid chromatography coupled to UV-DAD detector (Agilent 1200 series, Milan, Italy), set at wavelength to 283 nm, was used for the determination of 5-hydroxymethylfurfural (HMF). Next, 1.2 g of minced bread were mixed with 7 mL of distilled water and centrifuged at 25 °C at 5000 rpm for 10 min. The supernatant was collected and added to 1.5 mL of Carrez I (15% *w*/*v*), and 1.5 mL of Carrez II (30% *w*/*v*) reagent to precipitate the protein fraction. Then it was centrifuged at 5000 rpm for 10 min and 25 °C, and filtered through a Nylon filter 0.45 µm × 25 mm. This procedure was repeated in triplicate for C and PB on fresh bread (D_0_).

The chromatographic separation of the analytes was carried out by means of a Phenomenex, Luna C18 (2) 100Å 5 μm, (250 × 2 mm) operating at a flow of 0.500 mL/min. The elution was performed by combination of eluent A (H_2_O/HCOOH 95:5) and eluent B (acetonitrile/HCOOH 95:5) according to the following gradient: from 0 to 2 min, % B increased from 10 to 20 and held until 6 min; from 6 to 7 min % B was raised up to 100 holding for 5 min. Finally, the column was reconditioned for 5 min.

For the quantitative determination, a calibration curve was built by using five levels of standard concentration of the standard in the range between 0.06 mg/L and 0.2 mg/L; after checking the linearity range, the limits of detection and quantitation were evaluated (LOD = 0.02 mg/L; LOQ = 0.06 mg/L)”. All solutions were analyzed in triplicate.

### 2.7. Sensory Analysis

A pairwise panel test was done with 25 non-trained panelists (age range from 20 to 56 and 15 females/10 males) to assess differences in terms of aspect, pores, consistency, taste, and flavor and overall assessment on bread cubes. Each sample was identified by a three-digit code. The participants were asked to refrain from eating, smoking, drinking, or chewing gum for 1 h prior to testing. They were asked to give a score on a 9-point hedonic scale from 1 (dislike extremely) to 9 (like extremely) for several descriptors (aspect, crumb grain, texture, taste, and overall appeal) and the possibility to write free comments.

### 2.8. Statistical Analysis

Means and standard deviations calculated with SPSS (v. 26.0, SPSS Inc., Chicago, IL, USA) statistical software were used to perform one way (ANOVA) with a Tukey-Kramer post-hoc test to evaluate the effect of storage at a significance level of 0.05 (*p* < 0.05). A *t* test (*p* ≤ 0.05) was also performed to analyze differences between control and parchment-supplemented bread at the same day of storage.

## 3. Results and Discussion

### 3.1. Green Coffee Parchment Characteristics

The centesimal composition of green coffee parchment showed a high presence of fiber (64.3 ± 3.2 g/100 g), followed by protein (17.4 ± 2.1 g/100 g), ash (6.3 ± 1.7 g/100 g), and lipid (4.1 ± 0.3 g/100 g) with a moisture content of 7.9 ± 1.6 g/100 g, while the pH of green coffee parchment was about 6.6 ± 0.2. The high presence of fiber and the substantially low fat content make this by-product an interesting ingredient for fiber fortification in gluten-free bread production. Hydration properties showed the following values: *WBC* 4.49 ± 0.17 g/g, *WHC* 5.44 ± 0.41 g/g, *WAI* 11.94 ± 3.06 g/g, *WSI* 0.33 ± 0.13 g/g. Obtained hydration properties are very similar to those reported for coconut residues [20] that presented a very similar total fiber content and a predominant insoluble fraction. Particularly, *WHC* values of coffee parchment was similar to wheat bran [21] with a possible encouraging application in bread.

### 3.2. Bread Physical Characterization

**Figure 1:**
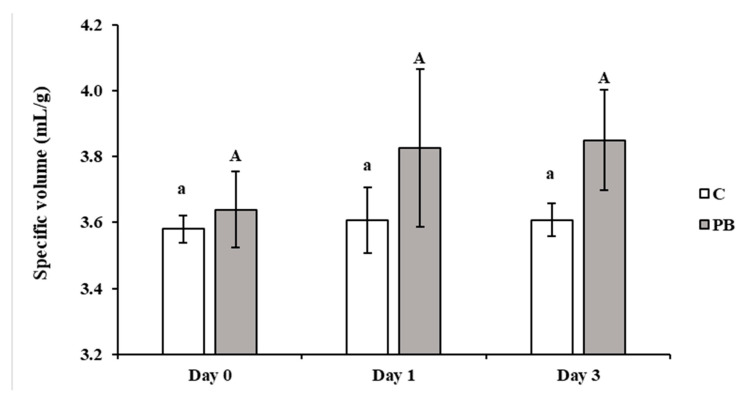
Specific volume (mL/g), mean and standard deviation, of control (C) and parchment supplemented (PB) bread loaves. Different small and capital letters, for C and PB, respec-tively, in-dicate significant differences (*p* ≤ 0.05) during storage.

The specific volume of bread loaves (Figure 1) was 3.6 mL/g both for C and P samples, being not affected by the presence of the green coffee parchment. In contrast with what was observed by Rinaldi et al. [13] for similar granulometries of cocoa bean shell addition to gluten-free bread, the specific volume was not influenced by the storage time remaining in the range 3.6–3.8 mL/g both in C and PB. Other authors [22] observed a significant volume increase with insoluble fiber addition to gluten-free bread, while Gómez et al. [23] evidenced a significant volume decrease for the addition of coffee-derived fiber in traditional wheat bread.

**Figure 2:**
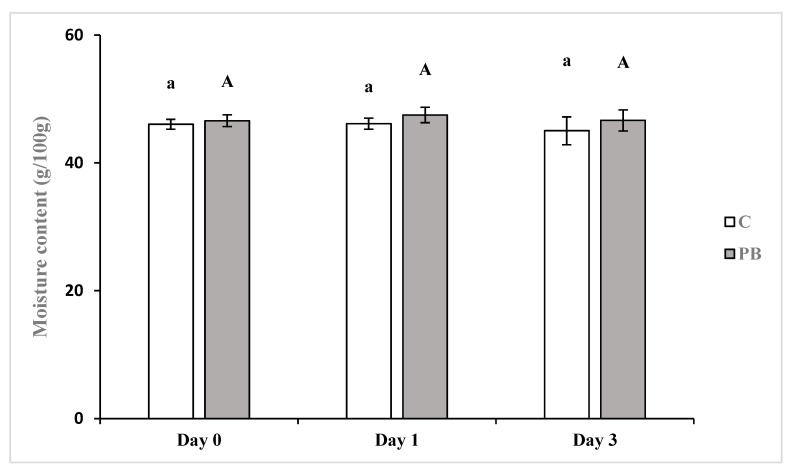
Moisture content (g/100 g), mean and standard deviation, of control (C) and parch-ment supplemented (PB) breads. Different small and capital letters, for C and PB, respec-tively, indicate significant differences (*p* ≤ 0.05) during storage.

Crumb moisture content (Figure 2) remained constant in PB and C (~46 g/100 g), maybe due to the considered short shelf life [13]. Considering that the moisture gradient between crumb and crust allows the migration of water between these two regions during storage [20], the packaging in alcohol-sprayed sealed air-tight plastic bags maybe have reduced this effect. No significant differences were evidenced between C and PB, due to the insoluble fiber of the green coffee parchment. In accordance with moisture content, water activity was in the range 0.94–0.95 for both samples during storage without no significant differences. This result is in accordance with the expected high content of insoluble dietary fiber of coffee parchment compared to other by-products that act as a physical binder of water without any effect on water activity [2].

**Figure 3:**
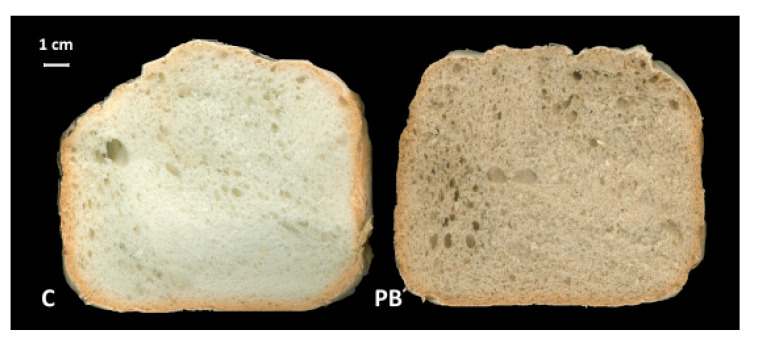
Appearance of control and parchment enriched breads.

**Figure 4:**
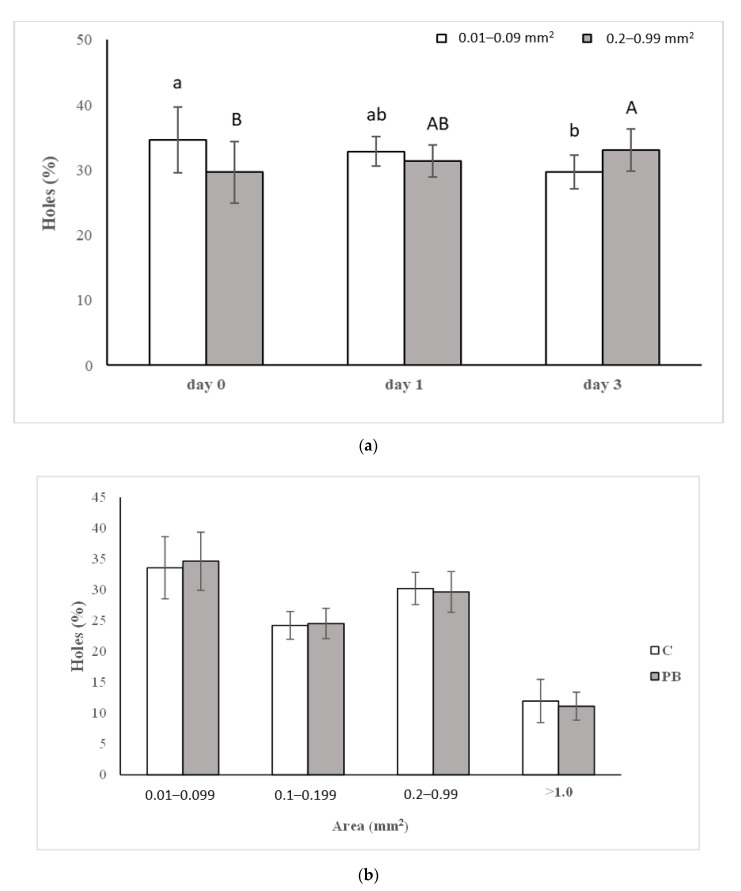
(**a**) Number of holes (%), mean and standard deviation, for the selected dimensional clasScheme 0. 099; 0.1–0.199; 0.2–0.99; >1.0) in fresh (day 0) control (C) and parchment bread (PB). (**b**) Number of holes (%) belonging to classes 0.01–0.09 mm^2^ and 0.2–0.99 mm^2^ in PB during storage. Different small and capital letters, for the two classes, respectively, are significantly different (*p* < 0.05).

The appearance of control and green coffee parchment-enriched bread was similar (Figure 3), confirming a similar percentage distribution of holes belonging to the four different dimensional classes (Figure 4a) in fresh bread (day 0). The most represented dimensional classes were 0.01–0.09 mm^2^ and 0.2–0.99 mm^2^ (~34% and ~30%, respectively), while the less represented one including largest holes, >1 mm^2^, showed a relative abundance of ~11%. The substantial similar sponginess together with the comparable specific volume of C and PB samples maybe evidenced that this kind of fiber did not interfere with thickening agents of the gluten-free mixture: guar flour and hydroxypropylmethylcellulose. In previous works [13,23] considering fiber from different origins, a significant decrease in the specific volume and substantial variation in the dimensional hole classes distribution, due to the competition for water exerted by different ingredients, was observed.

No significant differences were evidenced in C bread during storage concerning the % of holes for the considered dimensional classes. On the other hand, in PB, the 0.01–0.09 mm^2^ and 0.2–0.99 mm^2^ classes significantly varied during storage (Figure 4b). The decreasing % of holes belonging to the smallest class (0.01–0.09 mm^2^) in favor of the increasing % of them belonging to bigger one (0.2–0.99 mm^2^) was observable. This was probably due to the ability of parchment to bind water, hindering the formation and stabilization of small holes during bread production [24], but during storage, the holes’ walls were subjected to dehydration and got thinner by increasing their dimensions [25].

Colorimetric data are reported in Table 1. Crust and crumb PB lightness (L*) were not affected during storage but showed significant lower values than C bread even if the moisture content was comparable between the two samples (Figure 2). This was due to the natural darker color of parchment (in crust and crumb). The redness/greenness and yellowness/blueness parameters (a* and b*, respectively) were both influenced by the presence of coffee parchment especially in crumb: higher values (*p* < 0.001) were observed in supplemented bread, maybe due to the intrinsic color of the green coffee parchment. The general darkening of color parameters, due to the supplementation with this ingredient, could be very interesting to improve the gluten-free bread appearance that generally shows a pasty color [26].

Crust hardness (Table 2) resulted in 3.20 ± 0.89 N and 3.80 ± 1.11 N in C and PB, respectively, and as desired, no significant differences were observed in fresh breads (day 0). It decreased during storage in both samples, due to the water migration from crumb to crust favored by the absence of gluten [27], without significant differences. On the contrary, the evolution of crumb hardness presented a different trend (Table 2). Although during bread staling the loss of water and the starch retrogradation causes the hardening of crumb, the hardening of crumb at day 3 was observed in PB, while no significant differences were observed for C. The different behaviors in C and PB was maybe due to the interaction and competition between hydrocolloids of the mixture and the coffee parchment for binding water with a different redistribution of water between components in PB. In particular, a positive influence on the softness of the crumb during storage, by helping to retain moisture and by increasing the perception of crumb moistness, is expected for ingredients rich in soluble fiber [28], while coffee parchment is poor in this fraction. Moreover, if water is limited in the original mass, and many substances have to compete for it, high-fiber ingredients cannot carry out their functionality: this fact could explain the absence of the antistaling effect of coffee parchment [29].

Cohesiveness decreased in both samples during storage, as expected, without significant differences between samples. Springiness increased in C, while remaining constant in PB. Chewiness did not show variation in C during storage, while it decreased in PB. Conte et al. [29] underlined the correlation between these parameters and the crumb structure, in term of porosity, number of holes, and holes areas. This could explain the increasing hardness in PB, associated with a larger number of bigger holes and the smaller presence of smaller ones during storage.

### 3.3. Bread Chemical Characterization

Result of total phenolic content (TPC) was reported in Table 3: differently from what was expected [2], no significant differences were obtained in the TPC between C and PB, without scientific literature available to confirm or refute this results. It has to be underlined that phenolic compounds from different origins show different response to the Folin–Ciocalteu method, as well as the antioxidant capacity of each compound being affected by its structure [30].

On the other hand, the results obtained with the DPPH method, revealed that the antioxidant capacity of green coffee parchment-supplemented bread was more than six-fold higher with respect to control bread (Table 3). Therefore, we can assume that active compounds occur in the ingredient added to the supplemented bread, and they are able to react with the DPPH assay, consequently explicating a significant antioxidant capacity in the final product. In order to confirm this finding, the evaluation of oxidative stability was measured using the Oxitest reactor, an instrument able to record the effect of the stability of a sample submitted to accelerated oxidative conditions (by a combination of high temperature and oxygen pressure). This test permits to evaluate the total effect exerted by active compounds on the whole product, without any pre-treatment, rather than the action of selected extracted compounds on a specific reagent, as in the classic spectrophotometric assays.

The results recorded (Table 3), related to the induction period (expressed in minutes), showed values significantly higher in PB, about 50% higher than the control, confirming the presence of active compounds that are able to preserve the product during storage.

According to the results obtained by DPPH and Oxitest, the evaluation of the HMF showed a significant reduction of this compound in PB (3.9 ± 0.3 µg/g), since the amount of the analyte found was about two times lower than that measured in the control (8.3 ± 0.4 µg/g). This effect can be attributed to the presence of antioxidant compounds, as previously observed in buckwheat bread by a previous study [31], underlining the inhibitory effect of quercetin that is able to entrap HMF. The antioxidant capacity of flavonoids and vitamin C and E may also have a significant effect in reducing the progression of the Maillard reaction, as reported by previous studies [32].

### 3.4. Sensory Analysis

**Figure 5:**
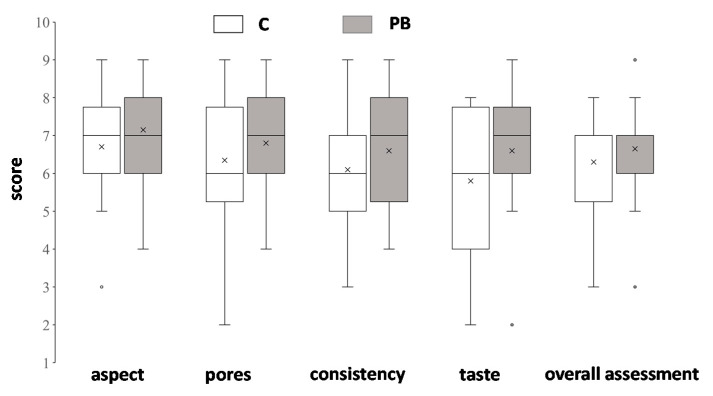
Box plot for sensorial analysis C and PB bread.

The sensory analysis evidenced that PB breads were perceived similar to the control for the majority of the considered descriptors (Figure 5): non-trained panelists caused also a great variability in judgments and absence of significant differences could be due to this fact. Among the free comments reported by panelists, PB was described with a better color and with more homogenous crumb characteristics than C. Interestingly, many panelists perceived PB samples as more salty than C, confirming the positive effect of green parchment on gluten-free breads that are generally recognized as bland and require high salt contents with related health problems. Only a few panelists gave the description of “bitter” (due to the presence of caffeine), but without a negative impact on the perceived taste.

## 4. Conclusions

This study attempts to evaluate for the first time in the literature the impact of the addition of green coffee parchment to gluten-free bread, in terms of structural, qualitative, and chemical properties during storage.

From the obtained results, it could be concluded that the green coffee parchment is a source of dietary fiber, able to improve the antioxidant capacity in gluten-free bakery products. The structure resulted less homogenous than the control and the staling during short-term storage seems to be accelerated but without a severe effect on crumb hardness and textural attributes. Further studies are required to understand the staling during a longer storage period. The color characteristics were appreciated by the sensory analysis, showing crust and crumb darkening, with promising expectations. Moreover, parchment-enriched bread showed a high antioxidant capacity, oxidative stability, and lower presence of HMF, as compared to the control bread.

In view of these considerations, the technological/functional aspects of green coffee parchment and the high global production of coffee, could promote the valorization of this by-product. Although the nutritional claim for fiber content was not reached for the supplemented bread studied in this work, the possibility of using this interesting by-product in other foods to obtain positive health, economic, and environmental sustainability effects could be considered.

Finally, this paper represents a preliminary study of GCP addition to bread and suggests that the debittering step could be a possible way to increase the amount of GCP in gluten-free bread in order to improve its nutritional and sensorial characteristics.

## Figures and Tables

**Table 1:** Crust and crumb colorimetric parameters for control (C) and green coffee parchment (PB) breads ^a^.

		*L* *			*a* *			*b* *		
		**C**	**PB**		**C**	**PB**		**C**	**PB**	
**Crust**	Day 0	86.88 ± 0.69 a	77.14 ± 1.03 a	***	2.48 ± 0.29 b	3.05 ± 0.28 b	***	14.33 ± 0.93 a	15.19 ± 0.85 ab	*
	Day 1	85.29 ± 0.72 b	77.24 ± 1.23 a	***	2.93 ± 0.32 a	3.22 ± 0.27 ab	*	15.12 ± 0.95 a	15.50 ± 0.81 a	
	Day 3	85.59 ± 1.13 b	77.04 ± 1.08 a	***	2.96 ± 0.45 a	3.27 ± 0.20 a	*	14.19 ± 0.86 a	14.88 ± 0.65 b	*
		*L* *			*a* *			*b* *		
		**C**	**PB**		**C**	**PB**		**C**	**PB**	
**Crumb**	Day 0	85.86 ± 0.74 a	69.11 ± 1.96 a	***	−0.66 ± 0.08 b	3.38 ± 0.24 b	***	11.68 ± 1.08 a	16.20 ± 0.74 a	***
	Day 1	83.74 ± 2.06 b	69.60 ± 1.22 a	***	−0.55 ± 0.09 a	3.32 ± 0.19 b	***	10.91 ± 0.81 a	15.91 ± 0.63 a	***
	Day 3	86.34 ± 1.28 a	70.12 ± 1.56 a	***	−0.63 ± 0.10 ab	3.67 ± 0.24 a	***	11.45 ± 0.46 a	16.27 ± 0.50 a	***

^a^ Means and standard deviations in column followed by different letters significantly differ (*p* < 0.05) among different storage times for the same bread. * *p* < 0.05; ** *p* < 0.01; *** *p* < 0.001.

**Table 2:** Crust and crumb textural parameters during storage for control (C) and parchment bread (PB).

	CRUST		CRUMB									
	Hardness (N)		Hardness (N)		Resilience (%)		Cohesiveness (-)		Springiness (-)		Chewiness (N)	
	C	PB	C	PB	C	PB	C	PB	C	PB	C	PB
Day 0	3.20 ± 0.89 a	3.80 ± 1.11 a	1.83 ± 0.23 a	1.79 ± 0.35 b	23.13 ± 1.59 a	25.52 ± 2.46 a	0.60 ± 0.01 a	0.62 ± 0.03 a	87.24 ± 3.55 b	87.36 ± 3.11 a	0.98 ± 0.17 a	0.96 ± 0.19 a
Day 1	1.83 ± 0.27 b	2.23 ± 0.57 b	2.01 ± 0.15 a	1.80 ± 0.38 b	22.02 ± 2.63 a	19.87 ± 3.42 b	0.56 ± 0.04 ab	0.52 ± 0.05 b	90.05 ± 2.76 b	84.55 ± 7.27 a	1.07 ± 0.20 a	0.80 ± 0.24 b
Day 3	1.54 ± 0.26 b	1.83 ± 0.51 b	1.88 ± 0.18 a	2.09 ± 0.72 a	24.52 ± 4.30 a	19.88 ± 2.87 b	0.56 ± 0.06 b	0.52 ± 0.05 b	95.62 ± 2.19 a	85.54 ± 3.59 a	0.96 ± 0.09 a	0.91 ± 0.27 ab

a,b Means and standard deviations in column followed by different letters significantly differ (*p* < 0.05) among different storage times for the same bread.

**Table 3:** Total phenolic content (TPC), antioxidant capacity, and oxidative stability, means and standard deviations, in control (C) and parchment bread (PB).

TPC (mg GAE/g)		Antioxidant Capacity (%)		Oxidative Stability (min)	
C	PB	C	PB	C	PB
0.98 ± 0.07	1.07 ± 1.16	10.9 ± 0.2	65.6 ± 1.6 *	493.0 ± 5.7	748.5 ± 12.0 **

* *p* < 0.05; ** *p* < 0.01.
